# Alterations in the Glycan Composition of Serum Glycoproteins in Attention-Deficit Hyperactivity Disorder

**DOI:** 10.3390/ijms24108745

**Published:** 2023-05-14

**Authors:** Kristína Kianičková, Lucia Pažitná, Paras H. Kundalia, Zuzana Pakanová, Marek Nemčovič, Peter Baráth, Eva Katrlíková, Ján Šuba, Jana Trebatická, Jaroslav Katrlík

**Affiliations:** 1 Institute of Chemistry, Slovak Academy of Sciences, SK-84538 Bratislava, Slovakia; 2 Department of Paediatric Psychiatry, Faculty of Medicine, Comenius University, The National Institute of Children’s Diseases, SK-83340 Bratislava, Slovakia

**Keywords:** glycosylation, ADHD, lectin-based glycoprotein microarray, MALDI-TOF mass spectrometry, biomarker

## Abstract

Changes in protein glycosylation are associated with most biological processes, and the importance of glycomic analysis in the research of disorders is constantly increasing, including in the neurodevelopmental field. We glycoprofiled sera in 10 children with attention-deficit hyperactivity disorder (ADHD) and 10 matching healthy controls for 3 types of samples: whole serum, sera after depletion of abundant proteins (albumin and IgG), and isolated IgG. The analytical methods used were a lectin-based glycoprotein microarray enabling high-throughput glycan analysis and matrix-assisted laser desorption/ionization time-of-flight (MALDI-TOF) mass spectrometry (MS) as a standard method for the identification of glycan structures. For microarray analysis, the samples printed on microarray slides were incubated with biotinylated lectins and detected using the fluorescent conjugate of streptavidin by a microarray scanner. In the ADHD patient samples, we found increased antennary fucosylation, decreased di-/triantennary N-glycans with bisecting N-acetylglucosamine (GlcNAc), and decreased α2-3 sialylation. The results obtained by both independent methods were consistent. The study’s sample size and design do not allow far-reaching conclusions to be drawn. In any case, there is a strong demand for a better and more comprehensive diagnosis of ADHD, and the obtained results emphasize that the presented approach brings new horizons to studying functional associations of glycan alterations in ADHD.

## 1. Introduction

Neurodevelopmental disorders are a widespread group of disorders that are classified under the Diagnostic and Statistical Manual of Mental Disorders, Fifth Edition (DSM-5, 2013) [1]. They often have a very early onset, and their pathophysiology depends on sex. These disorders share genetic, neural, hormonal, and immune mechanisms [2]. The group includes motor disorders, communication disorders, specific learning disorders, autism spectrum disorder (ASD), and attention-deficit/hyperactivity disorder (ADHD) [3]. All disorders that are included in the DSM-5 are connected with an environmental factor, genetic condition, or medical condition [4]. The prevalence of psychiatric disorders in childhood and adolescence is around 13% worldwide. ADHD is one of the most common behavioral disorders present in childhood that can continue into adulthood [5]. Worldwide prevalence of ADHD is ~5% in children and adolescents, and sometimes it is very hard to distinguish from ASD because there is some percentage of comorbidity (ADHD + ASD) [6]. ADHD is more often diagnosed in males; a higher male-to-female ratio is observed in children and adolescents [7], and the symptoms between males and females are different, which can hinder the diagnosis [8]. The main types of ADHD include being inattentive, hyperactive/impulsive, or showing a combined type of presentation. ADHD can affect many aspects of social life, including family, friends, school, and work relationships [9]. The symptoms of ADHD are very complex and involve problems with learning, inattention, and hyperactivity or impulsivity. The diagnosis of ADHD can be more difficult in some cases due to the overlap of symptoms with other neurodevelopmental disorders [10].

It is very hard and complicated to diagnose neurodevelopmental disorders correctly due to the necessity of objective quantification. A traditional diagnosis of ADHD is based on clinical observation and the preparation of questionnaires, screenings, rating scales, and interviews with the patient or patient’s parents; the diagnosis is not final after the first meeting, and it is very complex to consider in each case [11]. There are also imaging methods that research groups have been investigating for use in diagnosing ADHD, including electroencephalography (EEG) combined with deep convolutional neural networks [12,13] or structural and functional magnetic resonance imaging (MRI) [14,15,16]. The main effort, however, is devoted to the development of blood-based molecular and biochemical ADHD biomarkers. Among ADHD molecular biomarkers, researchers have studied microRNAs [17], methylation [18,19], and genetic sequencing [20]. In the case of biochemical biomarkers, several studies reported a connection between dopamine levels and ADHD manifestation [21,22]. Metabolic ADHD biomarkers, including fatty acids, amino acids, or sugars, were reported as well [23,24,25]. Protein biomarkers involve, for example, proteins associated with mitochondria [26], salivary α-amylase, and salivary secretory IgA and IgM [27].

The presented study sought to determine whether serum glycan analysis holds the potential to identify novel ADHD biomarkers based on changes in glycosylation. To our knowledge, only one study has been published so far regarding glycomic studies in ADHD. That work focused on the N-glycome of plasma samples by hydrophilic interaction liquid chromatography (HILIC) and weak anion exchange high-performance liquid chromatography (WAX-HPLC) methods and stated that ADHD is associated with increased antennary fucosylation of biantennary glycans and decreased levels of some complex glycans with three or four antennas. The authors of this study did not find any changes in the plasma glycome of ASD patients [28]. In our work, we applied combined glycan analysis using two different techniques, lectin-based glycoprotein microarray and matrix-assisted laser desorption/ionization time-of-flight MALDI-TOF MS methods [29,30], to study alterations in glycosylation in sera from ADHD patients and to search for possible glycan biomarkers of ADHD. Due to the need to improve the quality of ADHD treatment, there is still a strong demand for better and more complex diagnostics. Here, we emphasize that glycomic and glycoproteomic studies can bring new horizons to this field. In addition, more types of biomarkers and predictors need to be combined to improve diagnostic methods.

## 2. Results

### 2.1. Lectin-Based Glycoprotein Microarray Analysis

The lectin-based glycoprotein microarray analysis showed statistically significant differences in relative signal intensities between the ADHD and control groups in whole sera samples for 2 lectins: WGA (*p* = 0.032) and AAL (*p* = 0.005); in depleted sera (DS) for 4 lectins: MAL-II (*p* = 0.006), ConA (*p* = 0.044), RCA (*p* = 0.021), and PHA-E (*p* = 0.0008); and in IgG fractions for 2 lectins: SNA (*p* = 0.030) and AAL (*p* = 0.015) (Table 1). Signal-to-noise ratio (SNR) for MAL-I in sera samples was lower than 3, and these signals were not further analyzed.

**Figure 1:**
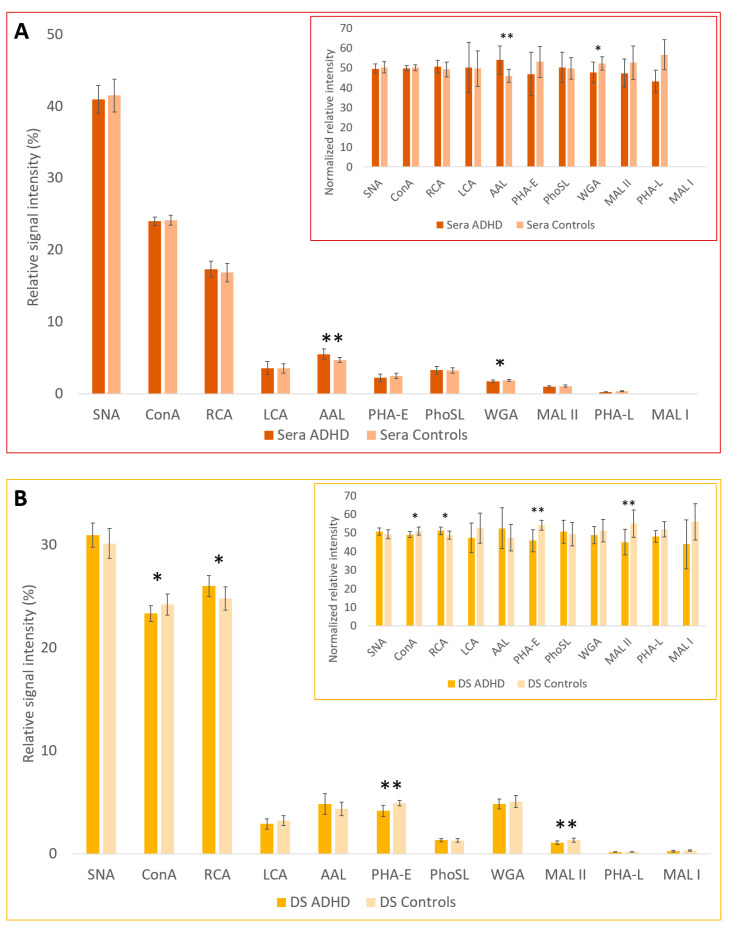

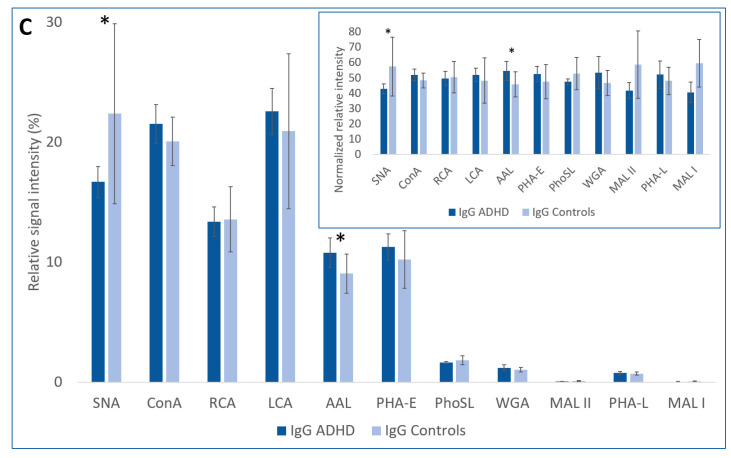
Graphic representation of relative signal intensities and normalized relative signal intensities (inserted figures) of interactions for the (**A**) sera, (**B**) DS (depleted sera without albumin and IgG), and (**C**) IgG fraction with lectins measured by the lectin-based glycoprotein microarray. Statistically significant differences (*t*-test, ** *p* < 0.01, * *p* < 0.05) are shown between the ADHD and control groups.

These results are graphically displayed in Figure 1. To better illustrate the low signals for some lectins, inserted figures show the normalized relative signal intensities calculated as a proportion of the signals for the control group and the ADHD group for each lectin, with the total signal value (control group + ADHD group) for each lectin having a value of 100.

### 2.2. MALDI-TOF MS Analysis

**Figure 2:**
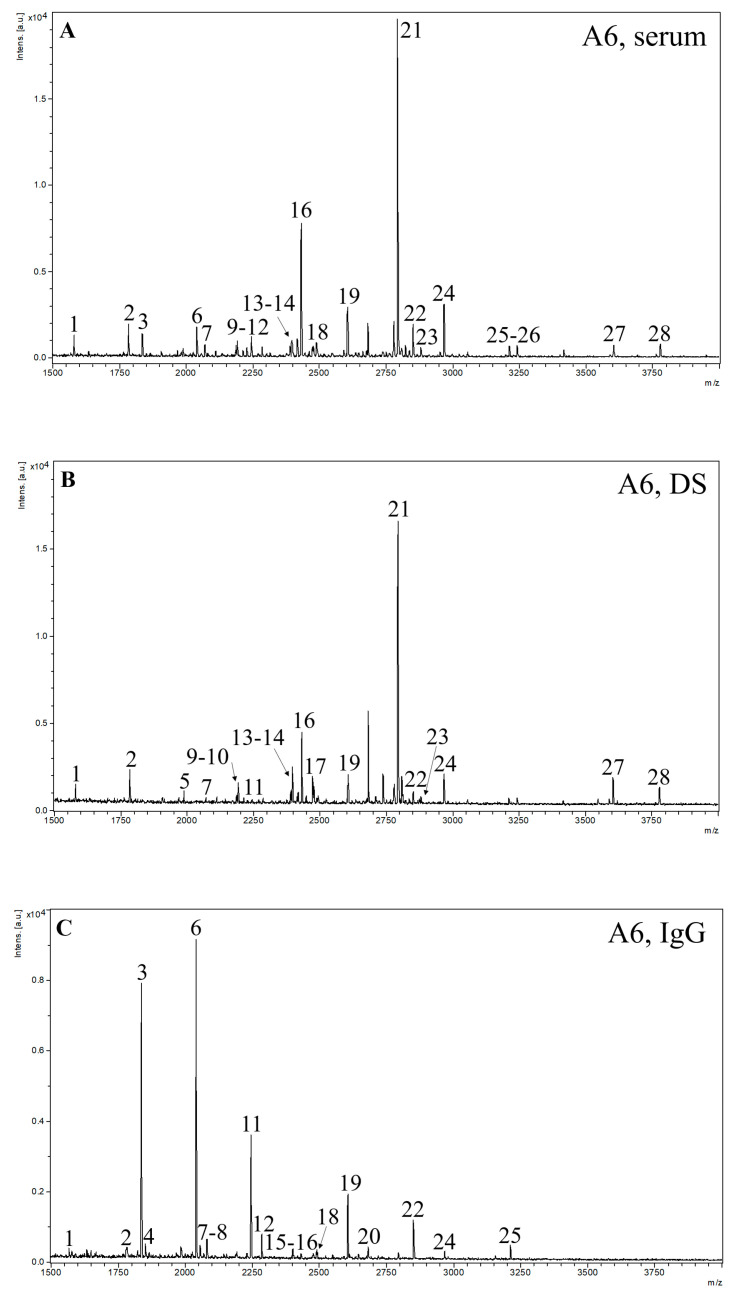
Representative MS spectra of samples labeled as A6 for (**A**) serum, (**B**) DS, and (**C**) IgG, with marked signals of N-glycans present in all spectra of individual groups of samples. The *m/z* range is 1500–4000.

The results obtained by the MALDI-TOF MS method are summarized in Table 2. Figure 2 illustrates the representative MS spectra for serum, depleted sera without albumin and IgG (DS), and IgG fraction samples of one ADHD patient, with marked signals of N-glycans present in all spectra of individual groups of samples. In Table 3 are shown the probable structures of N-glycans present in all spectra of individual groups of samples.

## 3. Discussion

From the results of the microarray analysis, we conclude that, in the sera samples from the ADHD patient group, the number of structures with antennary fucosylation (based on interactions with lectins AAL, PhoSL, LCA) is increased and the amount of N-acetylglucosamine (GlcNAc) and/or sialic acid (SA) structures (based on interactions with lectin WGA) is decreased. It can be assumed that the increased fucosylation is antennary and not core because signals belonging to the interactions with PhoSL lectin, recognizing only core fucosylation with the α1-6 linkage [31], and to the interactions with LCA lectin, with binding affinity to core-fucosylated, mono- and bi-antennary N-glycans [32], were not significantly different between the groups. Both of these fucosylations, core and antennary, are detectable by AAL, as this lectin containing five fucose binding sites can bind α1-3, α1-2, α1-4, and α1-6 linked fucose [33]. In the depleted sera of the ADHD patient group, we found a decrease in α2-3 sialylation (interactions with MAL-II), a decrease in mannose structures (interactions with ConA), a decrease in di-/triantennary complex type N-glycans with bisecting GlcNAc (interactions with PHA-E), and an increase in Gal/GalNAc structures (interactions with RCA). In IgG fractions, we found increased antennary fucosylation (interactions with AAL, PhoSL, and LCA, similarly as in the case of sera samples) and decreased α2-6 sialylation (interactions with SNA) in the ADHD patient group. There is no disagreement in those results with each other if all trends of differences in glycosylation within the individual sample types are also taken into account, though some of these trends do not meet a certain level of statistical significance. For example, the reactivity of sera samples with ConA was similar for both the control and patient groups; the reactivity of depleted sera with the same lectin was significantly higher for the control group; and the reactivity of the IgG fraction with ConA was lower, not even statistically significant, for the ADHD patient group.

Our findings, such as increased antennary fucosylation in the serum of ADHD patients, are in accordance with the main finding of another reported work [28] that focused on the N-glycome analysis of ADHD and ASD plasma samples, performed by different analytical methods (HILIC and WAX-HPLC).

Results of the MALDI-TOF MS analysis show statistically significant differences in the glycans H3N4F1 (*p* = 0.012) and H4N4F1 (*p* = 0.038) in sera samples, with both glycans upregulated in the ADHD patient group. The next structure in the N-glycan synthesis pathway, H5N4F1, is also upregulated in the ADHD group, although it was not statistically significant. All these N-glycans contain fucose in their structures. The H3N4F1 structure contains only a core fucose, while in the case of H4N4F1 and H5N4F1, they can also have isomeric structures containing antennary fucose. We detected the upregulation of fucose by the lectin-based glycoprotein microarray analysis as well. Specifically, by interaction with the lectin AAL (a statistically significant increase in sera samples of ADHD patients), which recognizes both antennary and core fucosylation, and with the lectins PhoSL and LCA (a similar trend but statistically insignificant), which recognize only core fucosylation, which is in agreement with previously published results on human plasma glycome in ADHD [28]. The next structure in the sera samples with a statistically significant difference is H5N4F1SA1 (*p* = 0.037), which is downregulated in ADHD samples. This structure has a more complex biantennary structure with both fucose and sialic acid (SA). To continue this study in the future, it would be valuable to focus on a detailed study of differentiation in the pathway of N-glycan structures, to add an MS protocol with linkage-specific sialic acid derivatization (a differentiation between the α2-3/2-6 SA isomers) to compare trends from a microarray with MS results focused just on the difference in sialylation, and to add an LC-MS method with a focus on core and antennary fucosylation.

When comparing the results of the microarray and MS analysis, we should emphasize that it is not possible to evaluate the contribution of a specific structure detected by MS analysis to the microarray analysis signal. Moreover, MALDI-TOF MS can only be considered a semi-quantitative method. The lectin-based glycoprotein microarray allows for screening only biologically available glycans on the surface of glycoproteins in comparison with MALDI-TOF MS, which analyzes all N-glycans in the sample after their enzymatic release [34]. All these factors must always be taken into account when comparing the data obtained by these two methods, and it is not possible to expect complete agreement between them.

As we mentioned, based on the microarray results of the serum samples of the ADHD patient group, fucose-containing structures are upregulated and SA-containing structures are downregulated, as evidenced by interactions with WGA, SNA, and MAL-II lectins (in the case of the last two, however, only as trends). Based on the MS results, H5N4F1SA1 is an example of a structure containing both SA and fucose that is downregulated in ADHD serum samples, and the structure with one additional SA (H5N4F1SA2) is an example of a structure containing both SA and fucose showing an opposite trend of upregulation in ADHD serum samples. This trend correlates with results from the MS analysis of DS samples, where statistically significant differences are represented by upregulation of structure H5N4F1SA2 (*p* = 0.006) in the ADHD group, and with results from analysis of the IgG fraction samples, where N-glycan structure H5N4F1SA1 (*p* = 0.022) is statistically downregulated as in the sera.

The other statistically significant downregulated structure in ADHD patients’ IgG fraction samples is the bisected N-glycan structure H5N5F1SA1 (*p* = 0.006), which can be formed by galactosylation of H4N5F1 and sialylation of H5N5F1, which are also downregulated in the IgG fraction of ADHD samples. Downregulation can also be seen in the structure H5N5F1SA2, which can be formed by the sialylation of H5N5F1SA1. The N-glycan pathway of this structure probably also has an impact on the statistically significant downregulation of α2-6 sialylation detected by the lectin-based glycoprotein microarray (lectin SNA), along with a trend in downregulation of α2-3 sialylation (lectin MAL-II). A statistically significant increase in fucosylation, indicated by interaction with lectin AAL in the microarray analysis, correlates with the trend in upregulation of fucosylated structures H3N4F1 and H4N4F1, in which, due to their high relative intensities in the MS spectrum, a significant proportion of the total response to fucose can be expected.

Fucosylated glycans have many important roles in the brain, such as memory or neuronal development. FUT8 (α1-6-fucosyltransferase) is responsible for core fucosylation in mammals. In a study focused on FUT8-knockout in mice that monitored their behavior and neurological activity, the results in FUT8-deficient mice indicated the presence of a schizophrenia-like phenotype, which manifested as a decrease in social interaction or enhanced locomotor activity [35]. Changes in behavior and neurological activity were also found in connection with congenital disorders of glycosylation (CDG) in a study of three individuals with pathogenic variants in *FUT8* [36]. It can be hypothesized that a decrease in core fucosylation can be one of the glycan signs of ADHD patients. 

Results of a study about SA and anti-ganglioside M1 antibody plasma levels in ASD patients showed a significant decrease in SA and higher positive anti-ganglioside antibody levels [37]. They concluded that there was a possibility of using SA as a biomarker for ASD. The mentioned research group later focused on the connection between neural cell adhesion molecules [38] and the gene *ST8SIA1* [39] with ASD, as they were both downregulated in ASD patients. Another group found an additional decrease in SA levels in the saliva of ASD patients and the potential impact of SA levels on the hyperactivity of ASD patients [40]. In those works, studying SA in connection with ASD, no correlation between the severity of ASD and SA levels was found. It was just hypothesized that gangliosides may contribute to higher sialylation in sera samples. However, as of now, there is no report on the connection between changes in sialylation and ADHD. Pivac et al. [28] observed no correlation between ASD or ADHD and SA levels. In this study, we found a decrease in sialylation in the IgG fraction of the ADHD patient group.

Other glycan structures, such as polysialic acids, gangliosides, and sialoglycans, are molecular components that have significant impacts on brain development, health, and diseases [41]. The proper function of gangliosides is connected with SA. There are studies that found a positive correlation between dietary SA supplementation in newborns and better cognition performance and intelligence. On the other hand, the negative impact of SA dysregulation can be seen in neurodegenerative disorders such as Alzheimer´s disease or Parkinson’s disease [42]. A study of a patient who had GM2-gangliosidosis and fulfilled the criteria for childhood disintegrative disorder found a decrease in sialylation in the cerebrospinal fluid [43]. Furthermore, it could be considered one of the potential connections between neuropsychiatric disorders and altered glycosylation. In addition, more types of biomarkers and predictors need to be combined to improve diagnosis.

## 4. Materials and Methods

### 4.1. Samples

Full blood samples were collected at the National Institute of Children´s Diseases in Bratislava, Slovakia. Ten blood samples were taken from children with a diagnosis of ADHD, and 10 blood samples were taken from healthy children without this diagnosis. The age of the children was between 9 and 19 years. The average age of the ADHD patients was 14.4 years, compared to 12.8 years for the healthy controls. Males made up 70% of the ADHD patients and 60% of the healthy controls. Both the patient and control groups had a healthy weight based on calculated body mass index (BMI) for age percentiles. Neither the patients nor the controls had any other acute inflammatory disease or any chronic disease apart from ADHD. Full blood samples were processed with the purpose of receiving three types of fragments: (i) sera; (ii) sera without IgG and HSA (depleted sera, DS); and (iii) IgGs. The study was approved by the Ethics Committee of the National Institute of Children’s Diseases and carried out in accordance with the Declaration of Helsinki and the Ethical Guidelines for Medical and Health Research Involving Human Subjects. Informed consent was obtained from all subjects involved in the study.

### 4.2. Sera Fragments

Whole blood samples were centrifuged in the original tubes of the BD vacutainer^®^ SST™ II Advance (Becton Dickinson, Franklin Lakes, NJ, USA) at 1500 rpm for 10 min at 20 °C. Serum was aliquoted into new microtubes and stored at −20 °C.

### 4.3. Sera Depletion

Multiple Affinity Removal Spin Cartridge HSA/IgG (Agilent, Santa Clara, CA, USA) was applied for this purpose. Samples were processed following the manufacturer´s protocol: they were diluted, the cartridge was prepared, the sample was applied twice through it, and flow-through was collected for later analyses. After each depletion, the cartridge was equilibrated according to the manufacturer´s protocol.

### 4.4. IgG Fraction

The IgG fraction was obtained using the Protein A column. First, 100 μL of the serum sample was diluted in 400 μL of wash/binding buffer (0.1 M sodium phosphate + 0.15 M NaCl, pH 7.4) and centrifuged. Then, 200 μL of protein A agarose resin (Jena Bioscience, Jena, Germany) was placed into an empty column of the Bio-Spin^®^ (Bio-Rad, Hercules, CA, USA), followed by the application of a diluted sample. In the next step, the column was washed with 10 column volumes of wash/binding buffer. Elution consisted of 5 column volumes using elution buffer (0.2 M glycine, pH 2.7). The first 3 column volumes were collected into clean microtubes pre-filled with 60 μL PBS, pH 8.5. Finally, 50 µL was saved for microarray (MA) analysis, and the rest of the volume was reduced using VivaSpin^®^ 500 centrifugal concentrators MWCO 10 kDa (Sartorius, Göttingen, Germany) and later used for MS analysis.

### 4.5. Protein Concentrations

For protein concentrations, we applied the DS-11 UV-Vis Spectrophotometer (DeNovix, Wilmington, DE, USA). The sera samples were diluted 10 times in PBS, while the depleted sera fractions (DS) and IgG fractions were measured directly without any dilutions. PBS was used as a blank for sera samples and IgG fractions, while BUFFER A from a kit was used as a blank for DS. All samples were measured 3 times.

### 4.6. Lectin-Based Glycoprotein Microarray

All types of samples were diluted in PBS (0.1 mg/mL), transferred into microtiter plates, and spotted (~1.2 nL per spot) in triplicates using the sciFLEXARRAYER S1 microarray spotter with appropriate piezo capillary PDC 80 (Scienion, Berlin, Germany) onto the epoxy microarray slides NEXTERION Slide E (Schott, Jena, Germany) into identical subarrays. The samples were spotted at 11 °C (the temperature of the microtiter plate) and 50% humidity. To immobilize the spotted samples, the slides were left overnight at room temperature with a humidity of 65%. Then, appropriate multi-well microarray slide masks (Grace Bio-Labs, Bend, OR, USA) were applied on the slides, and a solution of 3% BSA in PBS was applied onto the unreacted epoxy groups on the slide surfaces for 1 h at room temperature. The slides were washed with PBS containing 0.1% Tween-20 (PBST) and 11 biotinylated lectins listed in Table 4 (from Vector, Burlingame, CA, USA, except recombinant PhoSL, which was from the Korea Research Institute of Bioscience and Biotechnology, KRIBB, Jeonbuk, Republic of Korea) (25 μg/mL in PBST) were loaded into slide mask wells for 1 h at room temperature. The slides were then carefully washed with PBST, and streptavidin conjugated with fluorescent dye CF647 (Biotium, Fremont, CA, USA), 0.5 μg/mL in PBST, was loaded into slide mask wells for 30 min at room temperature in the dark. Slides were finally washed with PBST and distilled water, and the residual water was removed by centrifugation. Fluorescent signals in each spot were detected using an InnoScan^®^710 fluorescent microarray scanner (Innopsys, Carbonne, France) at an excitation wavelength of 635 nm. Software Mapix^®^ 7.4.1 (Innopsys) was applied for the analysis of signals. The fluorescence of each spot was measured and corrected to the background; the obtained signals were in arbitrary units (AU). The sum of the signals originating from the interactions of a particular sample with all 11 lectins was designated as 100%, and the proportions of signals for each of the lectins were expressed as the relative signal intensities. Signals for sample-lectin interactions with SNR < 3 were not analyzed. The differences between the studied groups (sera, DS, and IgG fractions) were determined by Student’s *t*-test (at *p* < 0.05 and *p* < 0.01). Relative signal intensities lower than 0.5% were not included in the statistical analysis of differences between the groups.

### 4.7. MALDI-TOF MS Analysis

The ADHD serum samples (10 µL) were supplemented directly with 40 µL of 10 mM Tris pH 7.5 buffer with 0.1% SDS. The DS (50 µL) were dried using Concentrator Plus, resuspended with 10 µL of Tris buffer, and supplemented with 40 µL of 10 mM Tris pH 7.5 buffer with 0.1% SDS. The IgG fraction samples were supplemented with Tris buffer to obtain 99.5 µL, and 0.5 µL of 20% SDS was added. The proteins were alkylated and reduced with 0.01 M dithiothreitol (DTT) and 0.025 M iodoacetamide (IAA). N-glycans were released by 1 μL of peptide-N-glycosidase (PNG-ase F; Roche Diagnostics, Basel, Switzerland). After overnight incubation at 37 °C, the released N-glycans were isolated using Supelclean ENVI-Carb SPE columns (Supelco/Sigma Aldrich, PA, USA) and lyophilized before permethylation. NaOH in the DMSO mixture was added in a volume of 150 μL to the lyophilized samples, and the permethylation was initiated by the addition of 150 μL iodomethane. After intensive mixing for 40 min at 2000 rpm at room temperature, the ice-cold water was added to interrupt the reaction. Chloroform was then used as an organic solvent for the extraction of permethylated N-glycans. Dried samples were dissolved in 50% methanol and placed onto MALDI-ground steel plates with an addition of 1 µL of 20 mg/mL DHB (in 30% ACN + 0.1% TFA + 1 mM NaOH) as the matrix solution. Spectra were acquired in positive ion and reflectron TOF mode by an UltrafleXtreme II MALDI mass spectrometer (Bruker, Billerica, MA, USA). MS data were interpreted manually using the software programs flexAnalysis 3.4 and Excel. N-glycan structures were interpreted by the GlycoWorkbench 2.1 software program. For the selection of N-glycans presented in the measured samples, more factors were applied: (i) available articles with a list of detected structures focused on sera and IgG permethylated N-glycome profiling with the application of MALDI-TOF MS [44,45]; (ii) the presence of particular N-glycan structures in all spectra; and (iii) signal-to-noise thresholds (S/N) ≥ 2. A list of structures detected in sera samples was also applied to the DS results. Finally, intensity values of the 21/18/17 N-glycan structures identified in the spectra of sera, DS, and IgG fragments were included in the statistics.

## 5. Conclusions

The presented results indicate upregulated fucosylation, downregulated sialylation, and a decrease in di-/triantennary complex type N-glycans with bisecting GlcNAc in the serum of ADHD patients, based on statistically significant differences in detected signals between ADHD patients and control samples. The increase in fucosylation, probably antennary, is confirmed by lectin-based glycoprotein microarray analysis with lectins AAL, PhoSL, and LCA for sera and IgG fraction samples and by MALDI-TOF MS analysis for structures H3N4F1 and H4N4F1 in sera samples. Downregulation of sialylation is demonstrated by lectin-based glycoprotein microarray analysis with lectin MAL-II for depleted serum and with lectin SNA for IgG fraction samples. The decrease in di-/triantennary complex type N-glycans with bisecting GlcNAc is assumed from lectin-based glycoprotein microarray analysis with lectin PHA-E for depleted serum samples. 

Few studies have been published so far on the topic of glycosylation changes in neurodevelopmental disorders, and only one of them addressed glycan changes in ADHD, with the results we obtained in agreement with this study. Moreover, some of our results are completely new because we used completely different analytical approaches, namely a combination of the lectin-based glycoprotein microarray and MALDI-TOF MS. The interest in researching ADHD and neurodevelopmental disorders in general at the molecular level is constantly growing, with the aim of better understanding the mechanisms of these disorders, their diagnosis, and treatment. Despite the limitations of our study due to the limited number of analyzed serum samples, ten from ADHD patients and ten from healthy controls, we believe that the study of glycan biomarkers has a lot to offer this field, and the approach and methodology we have chosen in this research have great potential.

## Figures and Tables

**Table 1:** Relative signal intensities of interactions for the sera, depleted sera (DS), and IgG fraction of ADHD and control samples with 11 lectins (and their simplified sugar specificities) measured by the lectin-based glycoprotein microarray. Statistically significant differences (*t*-test, ** *p* < 0.01, * *p* < 0.05) between the ADHD and control groups are shown in bold.

**Lectin**			SNA	ConA	RCA	LCA	AAL	PHA-E	PhoSL	WGA	MAL-II	PHA-L	MAL-I
**Sugar Specificity**			α2-6 SA	Man	Gal, GalNAc	Man (with Core Fuc)	Fuc	2/3 Ant. Bisecting CNG	Core Fuc	GlcNAc, SA	α2-3 SA	3/4 Ant CNG	α2-3 SA
**Relative signal intensity (%)**	Serum	ADHD	40.95	24.01	17.36	3.59	5.54 **	2.23	3.30	1.74 *	0.99	0.29	n.a.
		Controls	41.52	24.14	16.89	3.55	4.73 **	2.53	3.27	1.90 *	1.10	0.38	n.a.
	Depleted serum	ADHD	30.95	23.34 *	26.02 *	2.91	4.85	4.18 **	1.34	4.85	1.09 **	0.19	0.26
		Controls	30.15	24.22 *	24.81 *	3.24	4.38	4.93 **	1.31	5.08	1.34 **	0.20	0.34
	IgG	ADHD	16.69 *	21.52	13.37	22.57	10.80 *	11.28	1.66	1.21	0.07	0.78	0.06
		Controls	22.37 *	20.07	13.57	20.92	9.05 *	10.21	1.84	1.06	0.10	0.72	0.08

Ant—antennary; CNG—complex type N-glycans; Fuc—fucose; Gal—galactose; GalNAc—N-acetylgalactosamine; GlcNAc—N-acetylglucosamine; Man—mannose; SA—sialic acid; n.a.—value not available due to SNR < 3.

**Table 2:** Summary of relative intensities of individual N-glycan structures present in three types of samples (serum, DS, and IgG) in the ADHD and control groups, measured by MALDI-TOF MS. Statistically significant differences (*t*-test, ** *p* < 0.01, * *p* < 0.05) between the ADHD and control groups are shown in bold.

**Structure Nr.**			1	2	3	4	5	6	7	8	9	10	11	12	13	14
**Structure Notation**			H5N2	H6N2	H3N4F1	H4N4	H7N2	H4N4F1	H5N4	H3N5F1	H5N3SA1	H8N2	H5N4F1	H4N5F1	H6N3SA1	H9N2
**Relative signal intensity (%)**	Serum	ADHD	4.22	6.02	2.95 *	-	-	5.03 *	1.41	-	1.18	2.99	3.36	1.51	1.40	4.37
		Controls	3.37	5.88	1.88 *	-	-	3.23 *	1.40	-	1.16	3.12	2.64	1.30	1.51	3.32
	Depleted serum	ADHD	6.12	7.40	-	-	2.78	-	2.34	-	2.15	4.19	2.26	-	2.45	6.57
		Controls	7.41	8.23	-	-	3.04	-	2.32	-	2.35	4.32	2.29	-	2.61	5.75
	IgG	ADHD	0.72	1.22	21.36	0.95	-	33.45	1.03	3.56	-	-	17.09	6.12	-	-
		Controls	0.73	1.09	18.87	0.75	-	30.55	0.70	3.64	-	-	17.51	6.62	-	-
**Structure Nr**.			15	16	17	18	19	20	21	22	23	24	25	26	27	28
**Structure Notation**			H4N4F1SA1	H5N4SA1	H4N5SA1	H5N5F1	H5N4F1SA1	H5N5SA1	H5N4SA2	H5N5F1SA1	H6N5SA1	H5N4F1SA2	H5N5F1SA2	H6N5SA2	H6N5SA3	H6N5F1SA3
**Relative signal intensity (%)**	Serum	ADHD	-	14.74	-	1.37	5.71 *	-	31.08	3.24	0.84	4.98	1.37	0.72	0.87	0.64
		Controls	-	15.35	-	1.60	6.66 *	-	33.81	4.70	0.81	4.57	1.27	0.91	0.98	0.52
	Depleted serum	ADHD	-	11.06	4.53	-	4.50	-	29.92	2.27	1.74	4.98 *	-	-	2.54	2.19
		Controls	-	11.89	2.49	-	4.74	-	30.46	2.66	1.72	**4.02** *	-	-	2.10	1.59
	IgG	ADHD	0.92	0.77	-	1.92	5.60 **	0.78	-	3.12 *	-	0.61	0.77	-	-	-
		Controls	0.99	0.85	-	2.22	8.33 **	0.62	-	4.92 *	-	0.74	0.87	-	-	-

H—hexose (mannose or galactose), N—N-acetylglucosamine (GlcNAc), F—fucose, SA—sialic acid. Numbers present the number of sugar structures in the N-glycan structure.

**Table 3:** Probable structures of N-glycans present in all spectra of individual groups of samples.

Nr.	*m/z*	Structure Notation	Structure	Nr.	*m/z*	Structure Notation	Structure
1	1579.8	H5N2	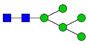	15	2401.2	H4N4F1SA1	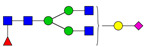
2	1783.9	H6N2	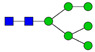	16	2431.2	H5N4SA1	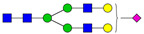
3	1835.9	H3N4F1	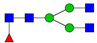	17	2472.2	H4N5SA1	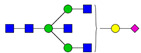
4	1865.9	H4N4	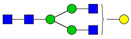	18	2489.3	H5N5F1	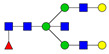
5	1988	H7N2	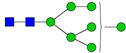	19	2605.3	H5N4F1SA1	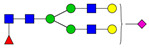
6	2040	H4N4F1	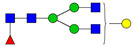	20	2676.3	H5N5SA1	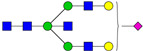
7	2070	H5N4	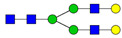	21	2792.4	H5N4SA2	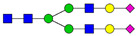
8	2081.1	H3N5F1	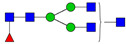	22	2850.4	H5N5F1SA1	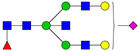
9	2186.1	H5N3SA1	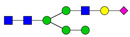	23	2880.4	H6N5SA1	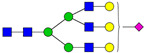
10	2192.1	H8N2	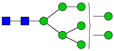	24	2966.5	H5N4F1SA2	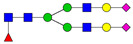
11	2244.1	H5N4F1	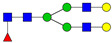	25	3211.6	H5N5F1SA2	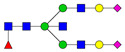
12	2285.2	H4N5F1	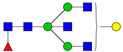	26	3241.6	H6N5SA2	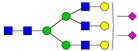
13	2390.2	H6N3SA1	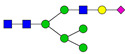	27	3602.8	H6N5SA3	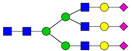
14	2396.2	H9N2	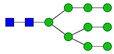	28	3776.9	H6N5F1SA3	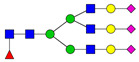

H—hexose, mannose (green circle) or galactose (yellow circle); N—N-acetylglucosamine (GlcNAc) (blue square); F—fucose (red triangle); SA—sialic acid (purple diamond). Structures were drawn in program GlycoWorkbench 2.1.

**Table 4:** The lectins used in the study and their sugar targets.

Lectin	Origin	Sugar Target
SNA	*Sambucus nigra*	α2-6 linked sialic acid
ConA	*Canavalia ensiformis*	Manα1-6Man, Manα1-3Man, Manα1-2Man, high mannose
RCA	*Ricinus communis*	Galβ1-4GlcNAc, GalNAc, Gal
LCA	*Lens culinaris*	αMan in N-glycans with core fucose, αMan in N-glycans
AAL	*Aleuria aurantia*	α1-3, α1-2, α1-4, α1-6 linked fucose
PHA-E	*Phaseolus vulgaris* (erythroagglutinin)	di-/triantennary complex type N-glycans with bisecting GlcNAc
PhoSL	*Pholiota squarrosa*	α1-6 linked fucose (core fucose)
WGA	*Triticum vulgaris*	GlcNAc, sialic acid
MAL-II	*Maackia amurensis* (hemagglutinin)	α2-3 linked sialic acid in O-glycans
PHA-L	*Phaseolus vulgaris* (leukoagglutinin)	tri/tetra-antennary complex type N-glycans
MAL-I	*Maackia amurensis* (leukoagglutinin)	α2-3 linked sialic acid in N-glycans

Gal—galactose; GalNAc—N-acetylgalactosamine; GlcNAc—N-acetylglucosamine; Man—mannose.

## Data Availability

Not applicable.
